# An Essential Signal Peptide Peptidase Identified in an RNAi Screen of Serine Peptidases of *Trypanosoma brucei*

**DOI:** 10.1371/journal.pone.0123241

**Published:** 2015-03-27

**Authors:** Catherine X. Moss, Elaine Brown, Alana Hamilton, Pieter Van der Veken, Koen Augustyns, Jeremy C. Mottram

**Affiliations:** 1 Wellcome Trust Centre for Molecular Parasitology, Institute of Infection, Immunity and Inflammation, College of Medical, Veterinary and Life Sciences, University of Glasgow, Glasgow, United Kingdom; 2 Medicinal Chemistry (UAMC), Department of Pharmaceutical Sciences, University of Antwerp, Wilrijk, Antwerp, Belgium; Federal University of São Paulo, BRAZIL

## Abstract

The serine peptidases of *Trypanosoma brucei* have been viewed as potential drug targets. In particular, the S9 prolyl oligopeptidase subfamily is thought to be a good avenue for drug discovery. This is based on the finding that some S9 peptidases are secreted and active in the mammalian bloodstream, and that they are a class of enzyme against which drugs have successfully been developed. We collated a list of all serine peptidases in *T*. *brucei*, identifying 20 serine peptidase genes, of which nine are S9 peptidases. We screened all 20 serine peptidases by RNAi to determine which, if any, are essential for bloodstream form *T*. *brucei* survival. All S9 serine peptidases were dispensable for parasite survival *in vitro*, even when pairs of similar genes, coding for oligopeptidase B or prolyl oligopeptidase, were targeted simultaneously. We also found no effect on parasite survival in an animal host when the S9 peptidases oligopeptidase B, prolyl oligopeptidase or dipeptidyl peptidase 8 were targeted. The only serine peptidase to emerge from the RNAi screen as essential was a putative type-I signal peptide peptidase (SPP1). This gene was essential for parasite survival both *in vitro* and *in vivo*. The growth defect conferred by RNAi depletion of *SPP1* was rescued by expression of a functional peptidase from an RNAi resistant *SPP1* gene. However, expression of catalytically inactive SPP1 was unable to rescue cells from the SPP1 depleted phenotype, demonstrating that SPP1 serine peptidase activity is necessary for *T*. *brucei* survival.

## Introduction

Human African Trypanosomiasis (HAT) is a disease of the developing world that is fatal if left untreated. Following strong collaborative efforts to combat the disease the number of new cases reported has dropped to 17,500 annually [1], although the actual case-load is likely to be at least three times the reported figure. While the improved situation is encouraging, there is still a pressing need for new chemotherapies for HAT. The current set of 4 licenced drugs (pentamidine, suramin, melarsoprol, and eflornithine) has problems with toxicity, mode of administration, or efficacy. Moreover the reduction in HAT prevalence has raised concerns of disease resurgence as complacency causes a withdrawal of interest and resources.

The publication of the genome of *Trypanosome brucei* [2] marked a leap forward in the quest for novel drug targets against this parasite. A combination of this genomic data together with systems to induce loss-of-function using RNA interference in *T*. *brucei* led to an explosion of studies highlighting specific genes that are important for the parasite and are thus potential drug targets, reviewed in [3]. Most recently, high throughput studies have screened large cohorts of genes for potential drug targets, taking in an entire chromosome [4], a gene family [5] or the entire genome [6].

We have undertaken an RNAi study in which we scrutinise a biochemically related set of genes, the serine peptidases, as potential drug targets. The serine peptidases are deemed suitable drug target candidates as some appear to be important virulence factors in both *T*. *brucei* and *Trypanosoma cruzi* [7–9]. Oligopeptidase B (OPB) is released in to the bloodstream from both *Trypanosoma evansi* and *T*. *brucei* where, as there are no effective endogenous inhibitors, it proteolytically inactivates host peptide hormones [10, 11]. In addition to their likely involvement in host-parasite interactions, there is evidence suggesting that serine peptidases are drug targets. Two classes of serine peptidase inhibitor have anti-trypanosomal activity against *T*. *brucei* [12] and the currently used therapeutic drugs suramin, pentamidine and diminiazine inhibit *T*. *brucei* OPB. The mode of action of these drugs is not necessarily via serine peptidases, and serine peptidases were not amongst the 28 genes linked to suramin action in a genome scale RNAi screen [13]. Nevertheless, there is sufficient evidence that *T*. *brucei* serine peptidases are worth investigating as potential drug targets. Importantly, peptidases are biochemically tractable and are likely to be druggable. Amongst the many examples of peptidases that have been successfully developed as drug targets is the serine peptidase DPP IV. Human DPP IV is the target of inhibitors which are licensed for the treatment of type 2 diabetes [14].

Here, we have conducted a medium-throughput investigation into potential novel drug targets, comprising all serine peptidases in *T*. *brucei*. From the 20 putative serine peptidases in *T*. *brucei* we found only one that was essential for parasite survival, a putative type I-like signal peptidase.

## Materials and Methods

### Ethics statement

All animal procedures were undertaken in adherence to experimental guidelines and procedures approved by The Home Office of the UK government. All work was covered by Home Office Project Licence PPL60/4442 entitled “Molecular Genetics of Trypanosomes and Leishmania". Mice were euthanised by carbon dioxide inhalation, in accordance with the Animals (Scientific Procedures) Act 1986.

### Bioinformatics

All *T*. *brucei* TREU 927 predicted protein sequences were downloaded from TriTrypDB. To search for predicted serine peptidases based on overall sequence homology, these protein sequences were used in a BLASTP search against the MEROPS peptidase database [15]. This generated a list of 29 potential serine peptidases. Of these, 16 were already annotated as serine peptidases in GeneDB at the time of searching. The remaining 13 sequences were used in a BLASTP search for closest relatives in the non-redundant protein database. Of these, 10 were found to be more closely related to other families of hydrolases, such as esterases and lipases, and therefore unlikely to be peptidases. Three sequences (Tb927.8.5760, Tb927.9.12980, Tb927.6.1810), however, were found to have sequence homology with serine peptidases. To determine whether these were active serine peptidases or pseudopeptidases, we searched for active site residues. Serine peptidases possess a nucleophilic serine residue, which forms part of the catalytic dyad or triad. The genes Tb927.8.5760 and Tb927.6.1810 code sequences containing the catalytic residues serine, aspartic acid, and histidine, the catalytic triad possessed by S9 family serine peptidases. So, based on sequence homology and the presence of the catalytic triad, Tb927.8.5760 and Tb927.6.1810 appear to be S9 serine peptidases.

In addition to identifying unidentified serine peptidases, we also examined the 9 genes annotated as serine peptidases in GeneDB, but were not retrieved by the BLASTP search against the MEROPS database. When individual protein sequences were used in a BLASTP search of the MEROPS database, several of these sequences were found to be peptidases from families other than the serine peptidase family, or unlikely to be peptidases at all (Tb927.5.1870, Tb927.10.3520, Tb927.11.3870, Tb927.11.8350, Tb927.2.5980, Tb927.2.3030). The GeneDB annotation for these six genes has since been altered to remove them as putative serine peptidases. Three other genes annotated as serine peptidases appear to code for pseudopeptidases, as they bear sequence homology to the serine peptidase families S51 (Tb927.11.12610) and S54 (Tb927.9.8260 and Tb927.8.1810), yet lack the designated active site residues for that family. Elsewhere, one predicted rhomboid-like gene (Tb927.2.2500) has been identified that contains S54 family active site residues [16] and was added to the list of serine peptidases (Table 1).

**Table 1:** Comparison of serine peptidase RNAi screen with RIT-seq phenotypes.

Family	Gene ID	Gene name	RIT-seq phenotype^†^	RNAi cell line phenotype
S8 Subtilisin	Tb927.3.4230	subtilisin-like serine peptidase	Normal in all	Normal
	Tb927.11.3780*	subtilisin-like serine peptidase	Abnormal in BSF 6d and PCF	Normal
S9 Prolyloligopeptidase	Tb927.11.12850	oligopeptidase B	Normal in all	Normal
	Tb927.10.8020	prolyl oligopeptidase, putative	Normal in all	Normal
	Tb927.5.4300	prolyl oligopeptidase-like	Normal in all	Normal
	Tb927.10.6940 *	dipeptidyl peptidase	As for Tb927.10.6970	Normal
	Tb927.10.6970 *	dipeptidyl peptidase	Normal in all	Normal
	Tb927.7.4940 *	oligopeptidase B-like	Abnormal in BSF 3d and 6d	Normal
	Tb11.v5.0175 *	oligopeptidase B-like	As for Tb927.7.4940	Normal
	Tb927.9.10970	BEM46-like	Normal in all	Normal
	Tb927.1.4780	S9D serine peptidase	Abnormal in diff BSF	Normal
	Tb927.8.5760	hypothetical protein	Abnormal in BSF 6d	Normal
	Tb927.6.1810	hypothetical protein	Normal in all	Normal
S10 Carboxypeptidase	Tb927.10.1030	serine carboxypeptidase III precursor	Normal in all	Normal
	Tb927.10.1040	serine carboxypeptidase III precursor	As for Tb927.10.1030	Normal
	Tb927.10.1050	serine carboxypeptidase III precursor	As for Tb927.10.1030	Normal
S26 Signal peptidase	Tb927.10.4590	mitochondrial inner membrane signal peptidase	Normal in all	Normal
	Tb927.5.3220	signal peptidase type I	Normal in all	Abnormal
S54 Rhomboid	Tb927.2.2500	hypothetical protein	Normal in all	Normal
S59 Nucleoporin	Tb927.11.980	nucleoporin	Normal in all	Normal

* Identical coding sequences

^†^ 3d, 6d denote 3 and 6 days after induction of RNAi

### Cell culture

The *T*. *brucei* bloodstream form 2T1 cell line [17] was a gift from David Horn (LSHTM, London) and was cultured in HMI-11 (HMI-9 (Gibco), 10% v/v FCS (Gibco,10270), Penicillin (20Uml^-1^)/Streptomycin (20 μgml^-1^) (Sigma)), as described previously [17]. Selective drugs were used at the following concentrations where appropriate: 2 μgml^-1^ puromycin, 2.5 μgml^-1^ phleomycin (InvivoGen), 5 μgml^-1^ hygromycin B (Calbiochem) and 10 μgml^-1^ blasticidin (InvivoGen).

### Plasmid construction

An RNAi target fragment of 400–600 bp was identified for each gene using RNAit target selection software [18]. Target fragments were amplified from *T*. *brucei* genomic DNA with forward and reverse primers including AttB1 and AttB2 sites respectively (S1 Table). The constructs used for RNA interference were based on the tetracycline inducible stem loop RNAi vector pRPA^iSL^[17]. The plasmid has been modified to incorporate two pairs of AttP1 and AttP2 sequences, in opposing directions, flanking the toxic ccdB gene [5]. Two copies of the AttB1/AttB2 tagged RNAi target fragment were incorporated in opposing directions in to the plasmid in a single reaction using BP recombinase. Recombination of the RNAi fragments in to the vector was tested using a ccdB susceptible strain of *E*. *coli* to grow the plasmid.

Recoded *SPP1* was synthesised by Dundee Cell Products. The recoded *SPPI* sequence (*SPPI* ^*R*^) codes for the same amino acid sequence as *SPPI* but only shares 76.3% identity (S1 Fig.). All segments of identity between *SPPI* and *SPPI* ^*R*^ are less than 20 base pairs long. *SPPI* ^*R*^ was inserted in to the plasmid pGL2243 using XbaI and BamHI restriction sites, generating pGL2308. This plasmid is designed to constitutively express SPPI from the tubulin locus, with the addition of a C-terminal 6x HA tag (Jones, NG, unpublished). To express catalytically inactive SPPI, the active site serine (Ser84) was changed to glycine by mutating pGL2308, carrying the coding sequence for *SPP1*, using site directed mutagenic PCR with oligonucleotides OL4309 and OL4310, generating the plasmid pGL2319.

### Cell lines and transfection

RNAi plasmids (5μg) were digested with AscI to linearise the integration cassette, which was then purified with a QiagenPCR purification kit, ethanol precipitated and resuspended in 10μl sterile water. 2T1 cells were transfected using a Nucleofector instrument (Lonza), in the on the X01 setting, using the Amaxa Human T cell Nucleofector kit (Lonza). Cells that had integrated the RNAi cassette were selected using hygromycin (2.5μg ml^-1^). Clonal cell lines generated in this step were then tested for puromycin sensitivity, and puromycin sensitive clones retained for further analysis. To determine the RNAi phenotype, cells were induced with 1μg ml^-1^ tetracycline at a cell density of 1 x 10^5^ml^-1^ and counted at 24 hour intervals for 5 days. Two individual clonal cell lines were analysed for each target gene. For expression from recoded *SPP1*, 5μg of pGL2308 or pGL2319 (inactive mutant) were digested with AscI, and transfected as for the RNAi plasmids (above). Cells that had integrated the recoded *SPP1* expression cassette were selected for using blasticidin (10μgml^-1^). Blasticidin resistant clones were subjected to a second round of selection with tetracycline to induce RNAi. Clones that survived 3 days in the presence of 1μgml^-1^ tetracycline were tested for expression of SPP1 by Western blot with an anti-HA antibody (Roche).

### Activity of DPP8 inhibitors

The following compounds that target human DPP8 and DPP9 (and in some cases DPP IV), were synthesised as described previously: UAMC-00374, UAMC-00662 [19]; UAMC-00491 [20]; UAMC-00691, UAMC-00701, UAMC-00726 [21]; vildagliptin [22]; UAMC-0800 [23]. These were stored in DMSO at -20°C and diluted immediately prior to use such that the maximum DMSO concentration in the assay did not exceed 1%. The IC_50_ of inhibitory compounds against recombinant TbDPP8 was determined using a fluorometric assay in a 96-well flat-bottomed plate in 50 mM Tris/HCl pH 7.5, 100 mM NaCl. Compounds were doubly diluted to give final concentrations ranging down from 100 μM and Tb-DPP8 was added to the assay at the indicated concentrations. The hydrolysis of the fluorogenic substrate H-Gly-Pro-AMC (Bachem) was measured on an EnVision plate reader using excitation and emission wavelengths of 355 nm ad 460 nm respectively. Fluorescence was plotted against compound concentration and the IC_50_ values calculated using GraFit (Erithicus).

To test the effect of compounds on parasites, inhibitory compounds were doubly diluted in HMI-11 from 250 μM (final inhibitor concentration) in a 96-well plate (Costar). Bloodstream form *T*. *brucei* 427 was added at a density of 1 x 10^5^ cells ml^-1^in 200 μl HMI-11 in a 96-well plate. Parasites were incubated with compounds for 48 hours, after which 0.1 volumes of Alamar Blue (0.49 mM resazurin in PBS) were added. After incubation for a further 24 hours, fluorescence was read at λ_excitation_ 485 nm and λ_emission_ 620 nm in an Envision Plate Reader (Perkin Elmer). The EC_50_ was calculated using GraFit software.

### Quantitative PCR

Total RNA was purified from 2–5 x 10^7^ *T*. *brucei* cells using the RNeasy miniprep kit (Qiagen) according to manufacturer’s instructions. Genomic DNA was removed with RQ1 DNaseI (Promega) and first strand cDNA synthesised using Superscript III (Invitrogen) according to manufacturer’s instructions. Quantitative PCR was done with a 7500 Real-time PCR system (Applied Biosystems) and the 25 μl reaction contained approximately 0.5 μg cDNA, 7.5 pmol of each primer, and Sybr Green PCR Master Mix (Applied Biosystems). The primers used for all real time PCR reactions are listed in S2 Table. The temperature cycling regime was as follows: 50°C for 2 min, 95°C for 10 min, followed by 40 cycles of (95°C for 15 sec and 60°C for 1 min). Relative quantification was done using the comparative Ct method (2^-ΔΔCT^) within the 7500 System SDS software.

### In vivo RNAi

Groups of four ICR mice were infected with 5 x 10^5^ *T*. *brucei* RNAi cells by intraperitoneal injection. RNAi was induced in two mice from each group by giving mice water containing 0.2g/L doxycycline with 5% sucrose. Parasitaemia was checked daily by diluting tail vein blood in 0.83% ammonium chloride and counting parasites using a haemocytometer.

## Results

### Identification of serine peptidases

We found 20 individual genes representing serine peptidases from six families in the *T*. *brucei* genome (Table 1). Notably, representatives of the largest peptidase family, the S1 (chymotrypsin/trypsin) peptidases, are not found at all in *T*. *brucei*, nor any of the trypanosomatids [15]. The most abundant serine peptidase family in *T*.*brucei* is the S9 prolyl oligopeptidase-like family (reviewed in [23]), with nine members present in the genome. This family includes oligopeptidaseB, a peptidase found only in plants, bacteria and kinetoplastids. There are three genes encoding oligopeptidase B like peptidases in *T*. *brucei* (Tb927.11.12850, Tb927.7.4940, and Tb11.v5.0175), the latter two of which are identical gene sequences. There are also two identical tandem copies of a dipeptidyl peptidase 8 like gene (Tb927.10.6940 and Tb927.10.6970), which is also a member of the S9 family. Other S9 peptidases include two prolyl oligopeptidase-like genes (Tb927.10.8020 and Tb927.5.4300), a BEM46 like gene (Tb927.9.10970), and a glutamyl endopeptidase (Tb927.1.4780). In addition to these previously indentified S9 peptidases, we identified two new S9 family members (Tb927.8.5760 and Tb927.6.1810) by performing a BLAST search of the MEROPS peptidase database [15] using the entire *T*. *brucei* genome. The genome also contains two subtilisin (S8 family) genes (Tb927.3.4230 and Tb11.02.1280), three carboxypeptidase (S10 family) genes (Tb927.10.1030, Tb927.10.1040, Tb927.10.1050), two signal peptidase (S26 family) genes (Tb927.10.4590 and Tb927.5.3220), one nucleoporin (S59 family) gene (Tb11.03.0140) and one rhomboid (S54 family) gene (Tb927.2.2500). We also identified four potential pseudopeptidases, which have sequence similarity but lack predicted active site residues: an S9 BEM46-like pseudopeptidase (Tb927.9.12980), two S54 rhomboid-like pseudopeptidases (Tb927.9.8260 and Tb927.8.1810) and an S51 dipeptidase E-like pseudopeptidase (Tb927.11.12610).

### RNAi screen

**Fig 1:**
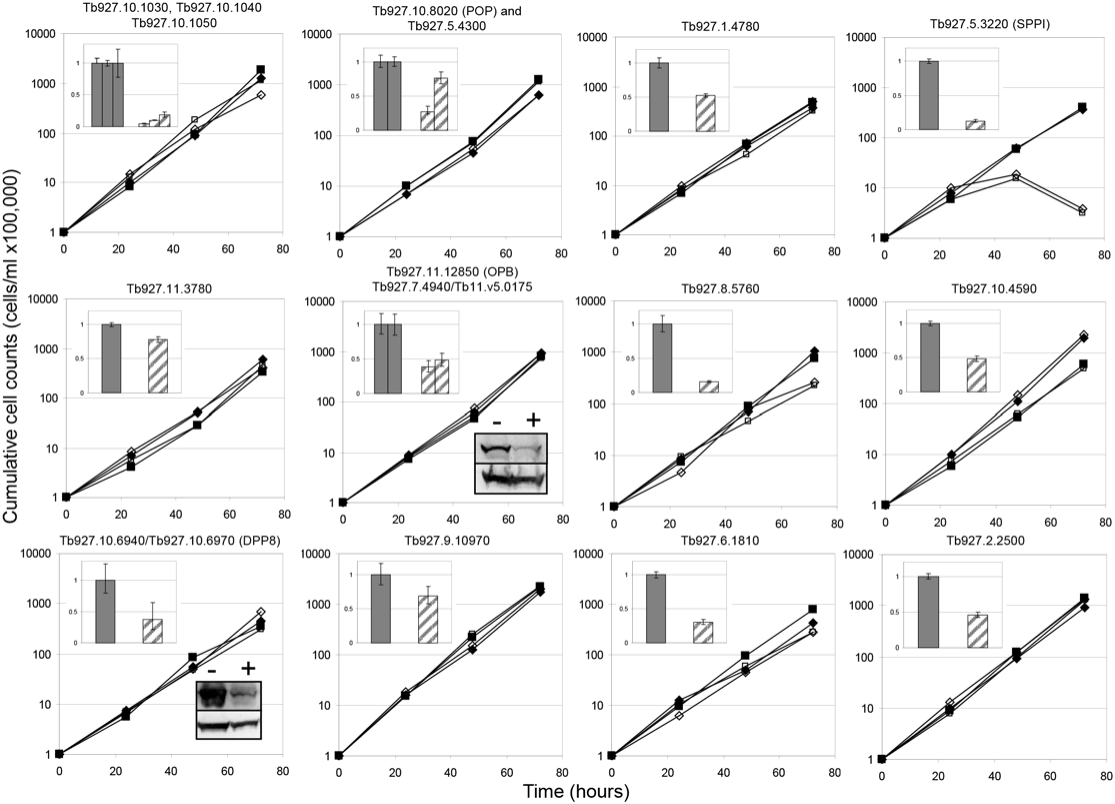
Effect of RNAi of serine peptidases on *T*. *brucei* cell growth *in vitro*. Two independent RNAi cell lines were induced with 1 μgml^-1^ tetracycline (open symbols) or left untreated (filled symbols). Cells were counted every 24 hours, diluted down to 1 x 10^5^ cells ml^-1^ and the cumulative cell number calculated. Insets: Target transcript levels in RNAi cell lines. Insets (upper left): Total RNA was isolated from non-induced (solid bars) or tetracycline-induced (striped bars) cells and used to make cDNA, which was used as a template in a real time PCR reaction using gene-specific primers (S2 Table). For cell lines in which multiple genes with non-identical sequences were targeted, PCR results for each target are shown. Results from three technical replicates are shown. Insets (lower right): For two targets, Western blots were done on lysates from control (-) or tetracycline induced (+) cells, using RNAi target protein-specific antibody (upper panel) or α-EF1-α (lower panel).

To determine the parasite’s requirement for individual peptidases, RNAi was used to knock down transcript levels. We chose to use an inducible stem loop RNAi vector, inserted at an optimised and tagged ribosomal locus [17] as this vector has been shown to have efficient and reproducible knock down of target genes [24]. The stem loop vector pRPa^iSL^ has been modified to include AttP1 and AttP2 recombination sites, enabling higher throughput cloning of RNAi target sequences [5]. After induction of RNAi with tetracycline, cell lines were monitored for five days for any growth defects. RNAi knock-down of all peptidases except one (Tb927.5.3220, a putative signal peptidase) had no effect on parasite growth (Fig. 1). Most serine peptidase genes were targeted individually, but some multi-target RNAi plasmids were designed. The tandem array of carboxypeptidase genes (Tb927.10.1030, Tb927.10.1040 and Tb927.10.50) share 84% identity, and so were targeted with a single RNAi fragment. There are also highly similar genes amongst the S9 serine peptidases. The non-contiguous genes coding for oligopeptidase B (OPB), (Tb927.11.12850), and OPB-like peptidases (Tb927.7.4940) share 40% identity, and may have similar activities and/or function. To overcome possible OPB/OPB-like redundancy, a knock-down construct targeting both genes was designed. As there is insufficient sequence identity to target both genes with a single RNAi fragment, a construct incorporating two tandem RNAi fragments was generated (pTL2078). Similarly there are two prolyl oligopeptidase (POP)-like genes, Tb927.10.8020 and Tb927.5.4300, having 34% identity, and these were simultaneously targeted with a dual RNAi construct (pTL2079).

The cell lines in which the OPB and OPB-like genes (Tb927.11.12850 and Tb927.7.4940/Tb11.v5.0175) or the POP and POP-like genes (Tb927.10.8020 and Tb927.5.4300) were targeted displayed no growth defect under RNAi. The non-essential nature of OPB seen in our RNAi lines is supported by the observation elsewhere that OPB knock-out cells had no loss of virulence [25].

To determine whether the correct gene was being targeted, we used quantitative PCR to measure relative levels of transcript. In all cases bar one (Tb927.11.980—omitted from dataset), there was a decrease in the amount of transcript 24 hours after RNAi induction, ranging from 20% to 90% depletion, with an average knock down of 60% (Fig. 1, inset). Antibodies against two target serine peptidases—OPB (Tb927.11.12850) and DPP8 (Tb927.10.6940 and Tb927.10.6970)—were available, and these were used to check targeted knock-down at the protein level. Significant knock down of both OPB and DPP8 was seen at 24 hours (Fig. 1 insets), supporting the quantitative PCR data.

### The S9 serine peptidases

**Fig 2:**
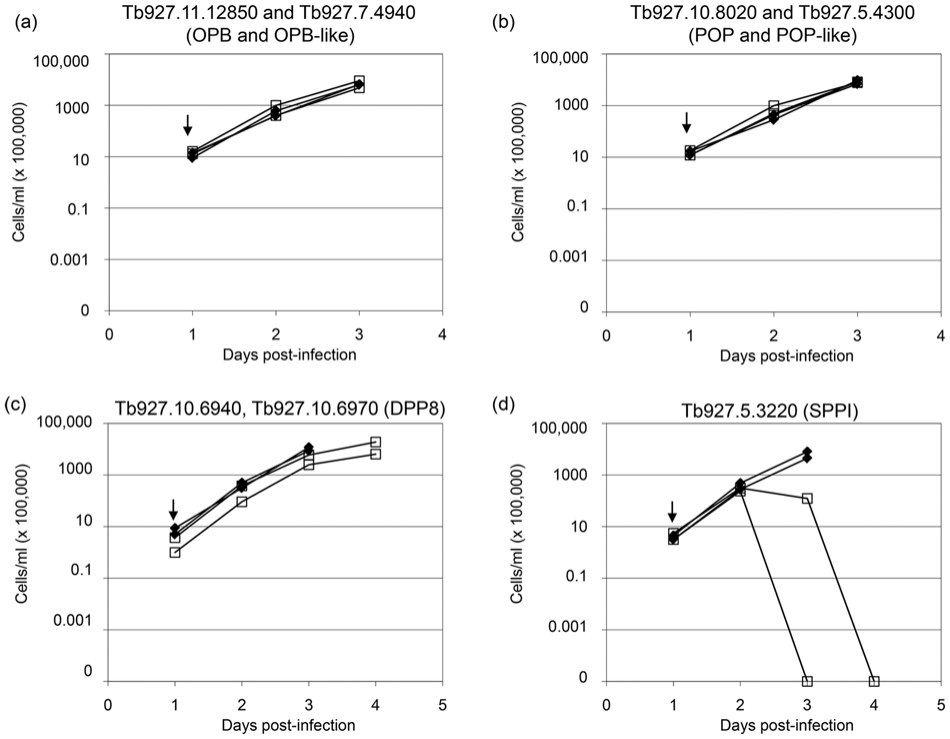
Effect of RNAi on *T*. *brucei* cell growth *in vivo*. ICR mice were inoculated with RNAi cell lines targeting the two OPB-like genes (a), two POP-like genes (b), dipeptidyl peptidase-8 (c), or the type-I signal peptidase (d). Two mice in each experiment were left untreated (filled symbols) and two were given doxycycline (open symbols) to induce RNAi. The arrow indicates doxycycline administration. Parasitaemia in infected mice was counted at the times indicated.

While OPB/OPB-like and POP/POP-like peptidases appear dispensable for the parasite *per se*, we investigated whether these peptidases might be involved in the host parasite interaction. Both OPB and POP are present in the secretome of *T*. *brucei gambiense* [26] and have been shown to be active in the plasma of infected rodents [7, 8], and could therefore influence the course of infection via cleavage of host peptides in the bloodstream. To test this, ICR mice were infected with RNAi cell lines targeting the two OPB/OPB-like genes (Tb927.11.12850 and Tb927.7.4940), or the two POP/POP-like genes (Tb927.10.8020 and Tb927.5.4300). Parasitaemia in infected animals was measured 24 and 48 hours after induction of RNAi (Fig. 2a and b). At these time points, there was no significant difference between induced and uninduced RNAi lines for either pair of peptidases. Animals were euthanised after this point, as parasiteamia levels rose above the humane threshold of 1 x 10^8^ cells ml^-1^. These results indicate that a reduction in either OPB/OPB-like or POP/POP-like peptidases has no effect on the progression of infection in mice, either individually or in concert.

**Fig 3:**
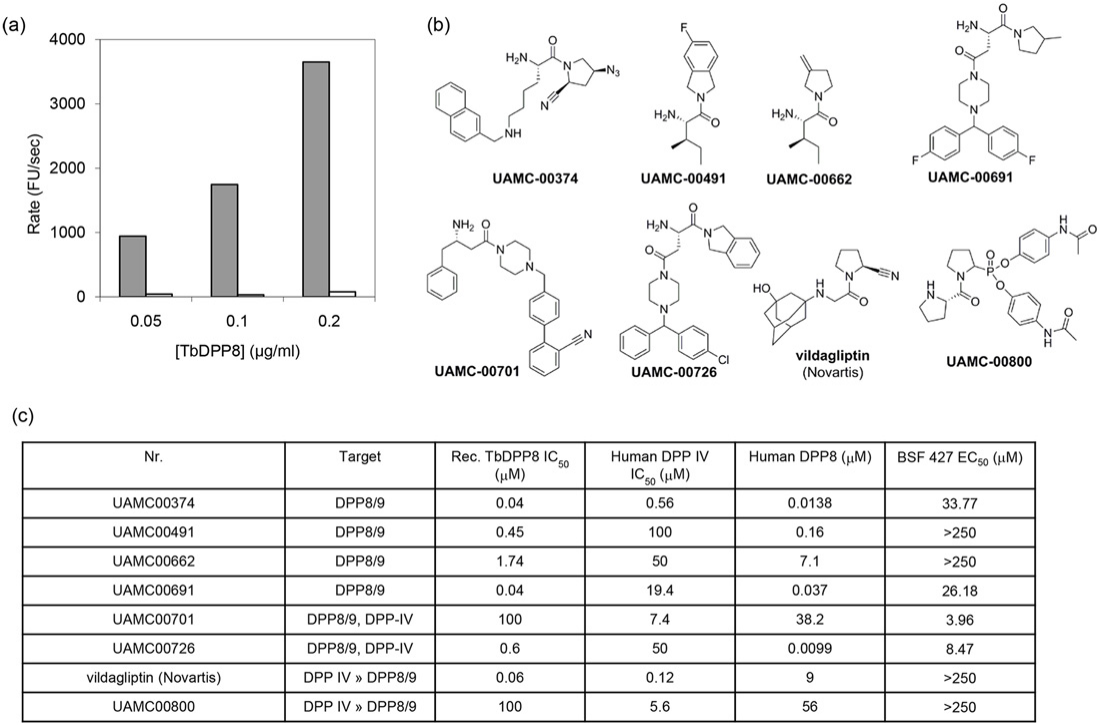
Recombinant TbDPP8 activity and inhibition. (a) Rate of cleavage by TbDPP8 of H-Gly-Pro-AMC (grey bars) and Z-Gly-Pro-AMC (white bars). (b) Structures of inhibitors used against TbDPP8. (c) Inhibitory activity of compounds against *T*. *brucei* DPP8, human DPP IV, human DPP8, and bloodstream form *T*. *brucei* 427.

We further investigated the *T*. *brucei* dipeptidyl peptidase (DPP8) for two reasons. Firstly, as a member of the S9 serine peptidase family, which includes putative virulence factors such as POP and OPB, we hypothesised that DPP8 in the host bloodstream may affect virulence. Indeed, the related human enzyme DPP IV is known to cleave, and thereby inactivate, circulating chemokines [27]. Secondly, human DPP IV is the target of type-2 diabetes drugs setting a precedent for the drugability of this enzyme. We expressed the recombinant enzyme, and confirmed that it is a Pro-specific aminopeptidase, as it was able to cleave the peptide substrate H-Gly-Pro-AMC, but not the N-terminally blocked Z-Gly-Pro-AMC (Fig. 3a). We used the recombinant DPP8 activity assay to test a panel of potential DPP8 inhibitors: one licensed diabetes drug, vildagliptin, and a series of compounds designed to target human DPP IV/8/9 enzymes [28] (Fig. 3b). *T*. *brucei* DPP8 was inhibited at 40–60 nM by UAMC00374, UAMC00691 and vildagliptin (Fig. 3c). These molecules, along with data on their inhibitory potency (IC_50_-values) against human DPP IV, DPP8/9, prolyl oligopeptidase, DPP II, and fibroblast activation protein (FAP) have been reported earlier [19–23]. The IC_50_ values for the human enzyme that are presented here were determined under slightly different conditions to those published earlier. This accounts for the slightly different, but nonetheless comparable IC_50_-values.

Nevertheless, these compounds were relatively ineffective against the bloodstream form of *T*. *brucei*, all having EC_50_ values greater than 25μM, suggesting that chemical depletion of DPP8 is not detrimental to the parasite. The most effective trypanocidal compounds (UAMC00701 and UAMC00726) were poor inhibitors of DPP8, further highlighting the lack of correlation between trypanocidal activity and DPP8 inhibition. The poor trypanocidal activity of DPP IV inhibitors is consistent with the result from the RNAi screen showing no effect of DPP8 knock down. In addition, RNAi of *DPP8 in vivo* caused no change in parasitaemia (Fig. 2c) indicating that, even if DPP8 does have activity in the host bloodstream, this does not affect the progression of infection.

### Type I signal peptidase

While GeneDB lists the *T*. *brucei* type I signal peptidase (SPP1, Tb927.5.3220) as a member of the *E*. *coli*-like S26A sub-family, we found it to be more similar to the animal type-I signal peptidases (S26B sub-family), bearing 33% sequence identity to human SEC11c compared with 12% with *E*. *coli* SP-1. In addition, *T*. *brucei SPP1* codes for a serine/histidine catalytic dyad, a feature of S26B peptidases, rather than the serine/lysine dyad found in S26A peptidases [20].

As SPP1 appears to be the only serine peptidase that is necessary for *T*. *brucei* survival *in vitro*, we examined more closely the effects of depleting this gene. Firstly, we tested whether the loss of fitness of the parasites held true *in vivo*. Mice were inoculated with the *SPP1* RNAi cell line and RNAi induced with doxycycline. Parasitaemia in the doxycycline treated mice dropped by 48 hours, and was undetectable by 72 hours (Fig. 2d). The kinetics of parasite depletion under RNAi *in vivo* is similar to that seen *in vitro*.

We looked for evidence that the phenotype displayed in the *SPP1* RNAi cell line was due to off-target transcript depletion, bymeasuring the level of a mitochondrial signal peptidase (mitSP, Tb927.10.4590) after induction of *SPP1* RNAi. The mitSP gene bears 42% identity to *SPP1*, yet should have insufficient local sequence similarity to be a target in the *SPP1* RNAi cell line. Quantitative PCR after RNAi of *SPP1* showed that, despite significant (>80%) reduction in *SPP1* transcript, mitSP transcript was not depleted. Indeed, mitSP transcript increased by over 50% (data not shown), possibly reflecting compensation for depletion of SPP1.

**Fig 4:**
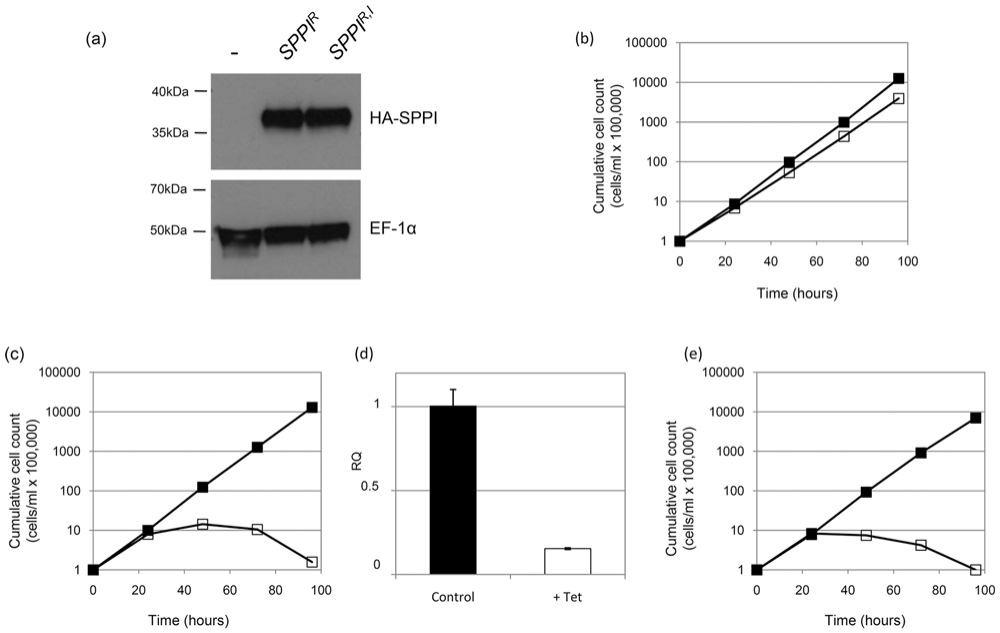
Rescue of *SPP1* RNAi growth defect by expression of recoded SPPI. (a) Expression of recoded HA-tagged SPP1 detected by Western blot. Cell lysates from RNAi cell line (-), the RNAi cell line expressing SPP1 from the recoded gene (*SPP1* ^*R*^), or the RNAi cell line expressing inactive SPP1 from a recodedgene (*SPP1* ^*R*,*I*^) were probed with anti-HA antibody (Roche). Detection of EF-1α was used as a loading control. (b, c and e) Parasite growth was measured in cell lines after inducing *SPP1* RNAi with tetracycline (open squares) or without treatment (closed squares) in the *SPP1* RNAi cell line expressing *SPP1* from: the recoded gene *SPP1* ^*R*^ (b), the parental *SPP1* RNAi cell line (c), or the RNAi cell line expressing inactive SPP1 from a recoded gene, *SPP1* ^*R*,*I*^ (e). (d) Quantitative PCR showing relative quantification (RQ) of endogenous *SPP1* transcript in the RNAi cell line expressing active SPP1 from the *SPP1* ^*R*^ gene either without induction (control, black bars) or after induction by tetracycline (+ Tet, white bars).

In order to verify that SPP1 knock-down was responsible for the loss of fitness in the RNAi line, we attempted to rescue the *SPP1* RNAi phenotype by expressing an RNAi-resistant recoded *SPP1* gene (SPP1^R^). *SPP1* ^*R*^ has insufficient sequence identity with the endogenous *SPP1* gene to be a target for the RNAi machinery and leads to constitutive expression of full length SPP1. We were able to detect expression of SPP1^R^ protein via its C-terminal HA tag (Fig. 4a). When *SPP1* RNAi was induced in this cell line, there was no growth defect (Fig. 4b), indicating that these cells had been rescued from the detrimental RNAi phenotype (Fig. 4c) by functional expression of SPP1. To verify that RNAi of endogenous *SPP1* remained functional in cells expressing SPP1^R^, we used quantitative PCR. Endogenous *SPP1* transcript was reduced by approximately 85% in tetracycline-induced cells, similar to knock-down levels seen in the parental *SPP1* RNAi line (Fig. 4d). This demonstrates that the normal phenotype in these cells was due to the presence of functional SPP1, rather than the emergence of cells refractory to RNAi. We next wanted to test whether the ability of the *SPP1* ^R^ gene to restore normal phenotype was due to its putative serine peptidase activity. The active site serine was mutated to glycine, and the expression of mutated SPP1confirmed by Western blot (Fig. 4a). Cells expressing catalytically inactive SPP1 displayed a growth defect when endogenous *SPP1* was down-regulated by RNAi (Fig. 4e), similar to that of the parental RNAi cell line (Fig. 4c).

With the aim of verifying the signal peptidase activity of *T*. *brucei* SPP1 we expressed and purified recombinant forms of the protein in *E*. *coli*. A small amount of soluble recombinant protein, lacking the predicted N-terminal transmembrane domain and carrying an N-terminal MBP fusion, was purified (not shown). However, we were unable to detect peptidase activity using a fluorogenic peptide substrate known to be cleaved by *E*. *coli* and *P*. *falciparum* signal peptidases [29]. We also attempted to rescue *E*. *coli* strains which are conditionally deficient in endogenous signal peptidase (LepB) [21], by expressing SPP1. Although expression of either full length or truncated SPP1 was successfully induced under conditions of LepB repression, this was insufficient to rescue the LepB deficient growth defect (not shown).

## Discussion

Technical developments, notably improved transfection efficiency [30], have expanded the scope of RNAi in *T*. *brucei*, to the extent that genome-wide RNAi screens can be carried out [6, 31]. A deficiency with screens on that scale is that, when the measured output is loss of fitness, clonal cell lines are not isolated from the genome-wide pool. Instead, regeneration of individual RNAi cell lines must be carried out to confirm the importance to viability of the identified genes. By exploiting recent developments in RNAi in *T*. *brucei* [5, 17] we have been able to screen the complete set of serine peptidases in *T*. *brucei*, followed up by qPCR validation of individual clones. In addition, we have been able to extend analysis of potentially interesting targets to an *in vivo* model.

We report that there are 20 serine peptidase genes in *T*. *brucei*, which represent 18 unique coding sequences. While only one gene, *SPP1*, emerged from our screen as essential, comparison with a genome-wide RNAi screen [6], showed that *SPP1* was not revealed by the RIT-seq method to be essential (Table 1). In contrast to the genome screen, we were able to validate each of the RNAi cell lines using quantitative PCR, and subsequently follow up our *in vitro* data with *in vivo* experiments. One explanation for the failure of RIT-seq to detect *SPP1* is the relatively small gene size. At 627bp, *SPP1* is likely to be under-represented in the RNAi library (which reported an average of >5 targets/CDS), and is possibly more likely to fall below the threshold of significant difference between induced and uninduced cells. Results for three other serine peptidases in our screen do not correlate with the RIT-seq data. One of these is the OPB-like gene (Tb927.7.4940) which displayed loss of fitness in bloodstream form cells after 3 and 6 days induction. However, in addition to the dual RNAi OPB/OPB-like cell line, we generated an RNAi cell line in which the OPB-like gene alone was targeted, which displayed no growth defect up to 4 days (data not shown). Altogether, a comparison of the two experiments illustrates the necessity of following up large-scale RNAi screens with independent validation of potentially interesting targets.

The S9 serine peptidases OPB and POP have been promoted as suitable drug targets [32] based on their presence in the host bloodstream, and their ability to cleave host peptides. However, these peptidases are not essential for parasite survival, at least *in vitro*. *T*. *brucei* OPB knockout parasites are viable and as virulent as wild-type parasites [25]. Similarly, *T*.*cruzi* and *L*. *major* OPB null parasites are viable, albeit compromised in their ability to infect host cells [3, 29]. By generating paired OPB and OPB-like RNAi knock-downs, we provide supporting evidence that OPB is non-essential, but also show that the related OPB-like gene is most likely not essential and that, if these two genes have an overlapping function, it is insensitive to down-regulation at the levels achieved in this screen. We have also found no evidence that OPB or POP influence parasite survival in host mice, so while they may be capable of cleaving host cell peptides, this capacity does not alter the course of infection.

SPP1 was the only serine peptidase in *T*. *brucei* demonstrated to be essential for parasite survival. Type-I signal peptidases have been shown to be essential in all bacterial species examined to date [13], and in yeast [33]. In *Leishmania major*, *SPP1* null mutants could not be generated, suggesting an essential function, and heterozygote mutants showed reduced infectivity [22]. *Plasmodium falciparum* dsRNA targeting type-I signal peptidase inhibited cell growth [29]. There is a high degree of conservation amongst type-I signal peptidase substrates, with the core A-X-A motif recognised by eukaryotic ER and bacterial type-I signal peptidases [34]. This substrate conservation is illustrated by the *in vitro* cleavage of *E*. *coli* MBP signal sequence by SPP1 from *Plasmodium falciparum* [29]. Type-I signal peptidases are not inhibited by standard serine protease inhibitors, and much work has gone in to developing signal peptidase inhibitors as broad spectrum anti-bacterials [19]. The SPP1 inhibitor arylomysin and its derivatives are effective against some but not all bacteria, a spectrum which is limited by natural mutations in SPP1 [35]. The effectiveness of arylomicin inhibitors on eukaryotic SPP1 is, to our knowledge, not known. As broad spectrum efficacy is not required for treatment of HAT, screening these S26A peptidase inhibitors against *T*. *brucei* would be an advisable approach.

We were able to show that *T*. *brucei* SPP1 is an active serine peptidase, and that this activity is essential for parasite survival. The failure to detect activity in recombinant SPP1 forms may be due to the absence of other signal peptidase complex subunits. A multi-subunit signal peptidase complex (SPC) is probably present in *T*. *brucei*, as other eukaryotic type-I signal peptidases are complexed with three other non-catalytic subunits [32]. We searched for similar non-catalytic signal peptidase complex subunits in *T*. *brucei* by carrying out BLASTP searches with yeast signal peptidase complex subunits against the *T*. *brucei* genome and retrieved one SPC3-like subunit (Tb927.5.1930). The SPC3 subunit is essential for the activity of the catalytic subunit (Sec11p) in yeast [36]. RIT-seq experiments indicate the *T*. *brucei* SPC-3 like subunit is essential [6], so it may be worth investigating this gene more closely to clarify whether it is an essential non-catalytic component of a *T*. *brucei* signal peptidase complex.

It is worth considering whether suitable SPP1 substrates are present in *T*. *brucei*. The cleavage motif for type-I signal peptidases (A-X-A) is well conserved across species, but the signal peptide also comprises a charged n-region and a hydrophobic h-region [34]. A search of the *T*. *brucei* genome for coding sequences containing a signal peptide (using SignalP) and an A-X-A motif within amino acids 22–32 found 230 genes (excluding pseudogenes) (S3 Table). It will be interesting to examine the processing and localisation of some of these potential SPP1 substrates within the *SPP1* RNAi cell line. On the basis of our data, a significant defect in ER transit would be predicted for many of these potential substrates.

## Supporting Information

S1 Table Primer sequences used to amplify RNAi target. (DOCX)

S2 Table Primers used for quantitative PCR. (DOCX)

S3 Table Potential SPP1 substrates. (DOCX)

S1 Fig Alignment of *SPPI* and recoded *SPPI* sequences. (DOCX)
