# Supplementary material for: An Essential Signal Peptide Peptidase Identified in an RNAi Screen of Serine Peptidases of Trypanosoma brucei
Source: PLoS One. 2015 Mar 27;10(3):e0123241. doi: 10.1371/journal.pone.0123241 (PMC4376731; doi:10.1371/journal.pone.0123241)
Supplement: S1 Table — (DOCX) [file pone.0123241.s001.docx]

**Supporting Information Table S1. Primer sequences used to amplify RNAi target**

|  | Gene-specific sequence | RNAi Plasmid |
| --- | --- | --- |
| Tb927.3.4230 | TLO101 ATGTAAAGTGGTGGCCAAGC  TLO102 AAAGACGACAACACGGAACC | pTL51 |
| Tb927.11.3780* | TLO103 CTCATTCGCTCTTCCTACCG  TLO104 AAAGGACGTGGTTTGATTGC | pTL52 |
| Tb927.11.12850* | OL2859 AGGACGTACTGAGCGGAGAA  OL2860 ACTCGCCTGTCGTAAGAGGA | pGL2120  pGL2078 (dual) |
| Tb927.10.8020 | OL2863 CATTTCCCAAACGTTCGACT  OL2864 CCTTTATCCACATCCCCCTT | pGL2063  pGL2079 (dual) |
| Tb927.5.4300 | OL2855 TACGGCGAATGTTGTGATGT  OL2856 CTCCCGGGGGAGGTATATTA | pGL2085  pGL2079 (dual) |
| Tb927.10.6940 | OL2966 AAGCGTTATCGTGTTGTCCC  OL2967 AGCACACTCTCCAGCCTGTT | pGL1888 |
| Tb927.10.6970 | As above | pGL1888 |
| Tb927.8.5760 | OL3876 CTTCGTTGTGGTGGGCTATT  OL3877 CCATTGCACAGATACGGTTG | pTL207 |
| Tb927.1.4780 | TLO105 GCCCCATCATTATTCACCAC  TLO106 AACTGTTACCGTAATGCCGC | pTL53 |
| Tb927.7.4940 | OL3511 gtaatgtgggagtctgccg  OL3512 acgtggaatctttggaaacg | pGL2078 (dual) |
| Tb11.v5.0175 | As above |  |
| Tb927.9.10970* | OL3943 tacttccccaacgtaccagc  OL3944 TTGTGTTGGGGATACCGAGTC | pTL210 |
| Tb927.6.1810 | OL3878 AACGATAGAGAACATGCGGG  OL3879 AAATATGAACGCAGGAACGG | pTL206 |
| Tb927.10.1030 | TLO107 CGTTAATCAATGGAGCGGAT  TLO108 GCTTTCCCCAACAACAAAGA | pTL54 |
| Tb927.10.1040 | As above | pTL54 |
| Tb927.10.1050 | As above | pTL54 |
| Tb927.10.4590 | TLO119 TCGGTGAATCGAAACCTACC  TLO120 CGGAGCAGTTGAAGGAAGAC | pTL60 |
| Tb927.5.3220 | TLO117 TGCACAAACAGGACCCATTA  TLO118 GTTTTGCCCATGAGTGGTCT | pTL59 |
| Tb927.11.980* | TLO127 ACCATTTTGGGAGCTACGTG  TLO128 CTTTTGTTTCAGCGTCGTCA | pTL64 |
| Tb927.2.2500 | TLO443 CACACTCTTAGAGCTCGGGG  TLO444 GCGGTAGCGAGTTTGAAGAC | pTL249 |

Forward TLO GGGGACAAGTTTGTACAAAAAAGCAGGCT-gene specific sequence

Reverse TLO GGGGACCACTTTGTACAAGAAAGCTGGGT-gene specific sequence
