# Supplementary material for: An Essential Signal Peptide Peptidase Identified in an RNAi Screen of Serine Peptidases of Trypanosoma brucei
Source: PLoS One. 2015 Mar 27;10(3):e0123241. doi: 10.1371/journal.pone.0123241 (PMC4376731; doi:10.1371/journal.pone.0123241)
Supplement: S2 Table — (DOCX) [file pone.0123241.s002.docx]

**Supporting Information Table S3. Primers used for quantitative PCR**

| Target Gene ID | Oligonucleotide Name / sequence |
| --- | --- |
| Tb927.3.4230 | OL4077 TGTGGCTCGGTTTGATAAGCT  OL4078 CGCCTGTCGGGCTATCC |
| Tb927.11.3780(one splice form of this gene)* | OL4079 CGGGACACGCGTTAACGT  OL4080 GCTCCACAGCCGTCTCAATT |
| Tb927.11.12850* | OL3001 CGTGCCGATCCCGAAGT  OL3002 GCGCTTCTCGTAATAATCCTTCTC |
| Tb927.10.8020 | OL3621 GCCACGGCATGGAGTGA  OL3622 CCAATCGCTTCCCTTGTCA |
| Tb927.5.4300 | OL4055 CAGCCTCCTCAGAGCATAGTGA  OL4056 TCGGTATTCTACACGCCCATACT |
| Tb927.10.6940 | OL2996 AAAGGCGGACAAAGCGTTATC  OL2995 CGGTGCAATGGCTGCAA |
| Tb927.10.6970 | As above |
| Tb927.8.5760 | OL4095 TGGAAGGCTCTGATATGGTGAA  OL4096 CACCAAAAGGCAGATGACAATC |
| Tb927.1.4780 | OL4081 GCTCGACACGGCTGCTAAA  OL4082 CGCAACGTCTACGGCATTT |
| Tb927.7.4940 | OL3709 GCACGCAGGTCCCCATAA  OL3710 CTCGAAATCCGTCGCTTCTT |
| Tb11.v5.0175 | As above |
| Tb927.9.10970* | OL3145 CTGGGTGGTGCAGTTGCTATT  OL3146 GCGATATGCTTCGCGTTGT |
| Tb927.6.1810 | OL4083 TGGACGCCCGGTAGCA  OL4084 TTCCCTGGGCACACGATT |
| Tb927.10.1030 | OL4085 TTTTTCTTATCTCGTTGCTACTCTTATTG  OL4086 GACGGCGGATAGCCTGAA |
| Tb927.10.1040 | OL4087 CCACTTTGCTCCGGCTACA  OL4088 ATATGCCTTCACCCTTTTTATTGC |
| Tb927.10.1050 | OL4089 CTGCTTGCATCCTGGTCGTA  OL4090 GTGCGAAGCGCTAGAGATAAACT |
| Tb927.10.4590 | OL4091 TTCTTACCATTACCGTCTTCACTACCT  OL4092 ACCATGTATGTTGTATGACCCTTCTC |
| Tb927.5.3220 | OL3958 ACGGCGCTGCGGATT  OL3959 TGCAGACATGGCTAATGATTCC |
| Tb927.11.980* | OL4093 GGCTTGCGGTTCGACAGTT  OL4094 CCATCCCGGCGGTAAAC |
| Tb927.2.2500 | OL4273 TGATTCCCACATGCATAGCAA  OL4274 GCAAAATGGACAAGCAAGCA |
