# Supplementary material for: An Essential Signal Peptide Peptidase Identified in an RNAi Screen of Serine Peptidases of Trypanosoma brucei
Source: PLoS One. 2015 Mar 27;10(3):e0123241. doi: 10.1371/journal.pone.0123241 (PMC4376731; doi:10.1371/journal.pone.0123241)
Supplement: S3 Table — (DOCX) [file pone.0123241.s003.docx]

**Supporting Information Table S4. Potential SPP1 substrates.**

| Tb05.5K5.430 | variant surface glycoprotein (VSG), putative,BAC from homologous region on chr5 |
| --- | --- |
| Tb08.27P2.110 | hypothetical protein, conserved,BAC from homologous region on chr8 |
| Tb08.27P2.260 | variant surface glycoprotein (VSG, atypical), putative,BAC from homologous region on chr8 |
| Tb09.v4.0051 | variant surface glycoprotein (VSG), putative,chrIX additional, unordered contigs |
| Tb09.v4.0073 | variant surface glycoprotein (VSG), putative,chrIX additional, unordered contigs |
| Tb09.v4.0088 | variant surface glycoprotein (VSG),chrIX additional, unordered contigs |
| Tb09.v4.0113 | variant surface glycoprotein (VSG, atypical), putative,chrIX additional, unordered contigs |
| Tb09.v4.0185 | variant surface glycoprotein (VSG), putative,chrIX additional, unordered contigs |
| Tb10.v4.0117 | variant surface glycoprotein (VSG), putative,chrX additional, unordered contig |
| Tb10.v4.0142 | variant surface glycoprotein (VSG), putative,chrX additional, unordered contig |
| Tb10.v4.0145 | variant surface glycoprotein (VSG, atypical), putative,chrX additional, unordered contig |
| Tb10.v4.0211 | variant surface glycoprotein (VSG), putative,chrX additional, unordered contig |
| Tb10.v4.0257 | variant surface glycoprotein (VSG), putative,chrX additional, unordered contig |
| Tb10.v4.0263 | variant surface glycoprotein (VSG), putative,chrX additional, unordered contig |
| Tb11.0660 | variant surface glycoprotein (VSG), putative,chrXI additional, unordered contigs |
| Tb11.1390 | hypothetical protein, conserved |
| Tb11.1450 | variant surface glycoprotein (VSG), putative |
| Tb11.1451 | variant surface glycoprotein (VSG), putative |
| Tb11.1490 | variant surface glycoprotein (VSG), putative,chrXI additional, unordered contigs |
| Tb11.v5.0118 | variant surface protein (VSG), putative |
| Tb11.v5.0139 | variant surface protein (VSG), putative |
| Tb11.v5.0155 | hypothetical protein, conserved |
| Tb11.v5.0208 | hypothetical protein, conserved |
| Tb11.v5.0213 | variant surface protein (VSG), putative |
| Tb11.v5.0216 | hypothetical protein, conserved |
| Tb11.v5.0219 | methyltransferase, putative |
| Tb11.v5.0226 | hypothetical protein, conserved |
| Tb11.v5.0284 | variant surface protein, atypical (VSG), putative |
| Tb11.v5.0287 | variant surface protein, putative |
| Tb11.v5.0299 | variant surface protein, putative |
| Tb11.v5.0311 | variant surface protein, putative |
| Tb11.v5.0317 | hypothetical protein, conserved |
| Tb11.v5.0338 | hypothetical protein, conserved |
| Tb11.v5.0415 | rRNA dimethyltransferase, putative |
| Tb11.v5.0444 | hypothetical protein, conserved |
| Tb11.v5.0447 | Variant Surface Glycoprotein, putative |
| Tb11.v5.0610 | variant surface glycoprotein (VSG, atypical), putative |
| Tb11.v5.0662 | variant surface glycoprotein (VSG), putative |
| Tb11.v5.0695 | variant surface glycoprotein (VSG), putative |
| Tb11.v5.0717 | hypothetical protein, conserved |
| Tb11.v5.0722 | variant surface glycoprotein (VSG)-related, putative |
| Tb11.v5.0781 | hypothetical protein, conserved |
| Tb11.v5.0873 | variant surface glycoprotein (VSG), putative |
| Tb11.v5.0916 | variant surface glycoprotein (VSG, atypical), putative |
| Tb11.v5.0923 | variant surface glycoprotein (VSG), putative |
| Tb11.v5.0928 | variant surface glycoprotein (VSG), putative |
| Tb11.v5.0935 | variant surface glycoprotein (VSG, atypical), putative |
| Tb11.v5.0948 | variant surface glycoprotein (VSG, atypical), putative |
| Tb11.v5.0952 | variant surface glycoprotein (VSG, atypical), putative |
| Tb11.v5.0953 | variant surface glycoprotein (VSG, atypical), putative |
| Tb11.v5.0958 | variant surface glycoprotein (VSG, atypical), putative |
| Tb11.v5.0974 | variant surface glycoprotein (VSG), putative |
| Tb11.v5.0979 | variant surface glycoprotein (VSG, atypical), putative |
| Tb11.v5.1009 | variant surface glycoprotein (VSG), putative |
| Tb11.v5.1018 | variant surface glycoprotein (VSG), putative |
| Tb11.v5.1023 | variant surface glycoprotein (VSG), putative |
| Tb927.1.1060 | hypothetical protein, conserved |
| Tb927.1.2730 | hypothetical protein, conserved |
| Tb927.1.5130 | hypothetical protein, unlikely |
| Tb927.1.5300 | variant surface glycoprotein (VSG), putative |
| Tb927.1.800 | hypothetical protein, unlikely |
| Tb927.10.10120 | hypothetical protein, conserved |
| Tb927.10.10220 | procyclin-associated gene 2 (PAG2) protein (PAG2) |
| Tb927.10.10250 | EP2 procyclin (EP2) |
| Tb927.10.10260 | EP1 procyclin (EP1) |
| Tb927.10.11140 | hypothetical protein, conserved |
| Tb927.10.11210 | hypothetical protein, conserved |
| Tb927.10.11930 | beta-D-hydroxybutyrate dehydrogenase |
| Tb927.10.12050 | hypothetical protein, conserved |
| Tb927.10.12450 | SNARE domain-containing protein, putative |
| Tb927.10.13430 | citrate synthase, putative |
| Tb927.10.13660 | hypothetical protein, conserved,hypothetical protein |
| Tb927.10.13830 | chaperone protein DNAj, putative |
| Tb927.10.15320 | hypothetical protein, conserved |
| Tb927.10.15830 | hypothetical protein, conserved |
| Tb927.10.16240 | Variant Surface Glycoprotein, putative |
| Tb927.10.16370 | Variant Surface Glycoprotein, putative |
| Tb927.10.16420 | variant surface glycoprotein, fragment |
| Tb927.10.16560 | variant surface glycoprotein (VSG), putative |
| Tb927.10.1920 | hypothetical protein |
| Tb927.10.2410 | MSP-C, putative (MSP-C) |
| Tb927.10.3620 | Laminin-like protein, putative |
| Tb927.10.4780 | GPI inositol deacylase precursor (GPIdeAc) |
| Tb927.10.5090 | hypothetical protein, conserved |
| Tb927.10.5400 | hypothetical protein, conserved |
| Tb927.10.6180 | hypothetical protein, conserved |
| Tb927.10.6640 | COP-coated vesicle membrane protein erv25 precursor, putative,ER--golgi transport protein erv25 precursor, putative |
| Tb927.10.7140 | membrane-bound acid phosphatase 2 (MBAP2) |
| Tb927.10.750 | hypothetical protein, conserved |
| Tb927.10.7890 | hypothetical protein, conserved |
| Tb927.10.9370 | hypothetical protein, conserved |
| Tb927.11.11380 | hypothetical protein, conserved |
| Tb927.11.11960 | hypothetical protein, conserved |
| Tb927.11.12230 | heat shock protein HslVU, ATPase subunit HslU, putative,ATP-dependent hsl protease ATP-binding subunit hslU, putative |
| Tb927.11.13090 | elongation factor 1 gamma, putative |
| Tb927.11.13190 | elongation factor 1 gamma, putative |
| Tb927.11.13440 | mitochondrial carrier protein (MCP21) |
| Tb927.11.13520 | hypothetical protein, conserved |
| Tb927.11.13620 | hypothetical protein, conserved |
| Tb927.11.13740 | receptor-type adenylate cyclase GRESAG 4, putative |
| Tb927.11.16070 | TFIIH basal transcription factor subunit (p34) |
| Tb927.11.16940 | hypothetical protein, conserved |
| Tb927.11.17110 | variant surface glycoprotein (VSG), putative |
| Tb927.11.17190 | variant surface glycoprotein (VSG), putative |
| Tb927.11.17320 | variant surface glycoprotein (VSG), putative |
| Tb927.11.17370 | variant surface glycoprotein (VSG), putative |
| Tb927.11.18250 | variant surface glycoprotein (VSG), putative |
| Tb927.11.1830 | hypothetical protein, conserved |
| Tb927.11.18330 | variant surface glycoprotein (VSG), putative |
| Tb927.11.18480 | variant Surface Glycoprotein, putative |
| Tb927.11.18840 | variant surface protein (VSG), putative |
| Tb927.11.18950 | variant surface protein (VSG), putative |
| Tb927.11.19180 | variant surface glycoprotein (VSG, atypical), putative |
| Tb927.11.19870 | variant surface glycoprotein (VSG, atypical), putative |
| Tb927.11.20100 | variant surface glycoprotein (VSG, atypical), putative |
| Tb927.11.20390 | variant surface glycoprotein (VSG), putative |
| Tb927.11.20500 | variant surface glycoprotein (VSG), putative |
| Tb927.11.20700 | variant surface glycoprotein (VSG, atypical), putative |
| Tb927.11.2670 | Nucleoporin (TbNup59) |
| Tb927.11.3780 | subtilisin-like serine peptidase,serine peptidase, clan SB, family S8-like protein |
| Tb927.11.4380 | ATP-dependent RNA helicase, putative,DEAD/DEAH box RNA helicase, putative |
| Tb927.11.4650 | hypothetical protein, conserved |
| Tb927.11.5060 | hypothetical protein, conserved |
| Tb927.11.6100 | hypothetical protein, conserved |
| Tb927.11.6150 | hypothetical protein, conserved |
| Tb927.11.6170 | protein transport protein SEC31, putative,cytosolic coat protein, putative |
| Tb927.11.6860 | hypothetical protein |
| Tb927.11.7490 | hypothetical protein, conserved |
| Tb927.11.7500 | hypothetical protein, conserved |
| Tb927.11.9560 | oxidoreductase, putative |
| Tb927.11.9580.1 | unspecified product |
| Tb927.2.5280 | trans-sialidase, putative |
| Tb927.2.6130 | ABC transporter, putative |
| Tb927.3.150 | variant surface glycoprotein (VSG), putative |
| Tb927.3.180 | variant surface glycoprotein (VSG, atypical), putative |
| Tb927.3.190 | variant surface glycoprotein (VSG), putative |
| Tb927.3.3890 | hypothetical protein, conserved |
| Tb927.3.4050 | hypothetical protein, conserved |
| Tb927.3.440 | variant surface glycoprotein (VSG), putative |
| Tb927.3.4740 | hypothetical protein, conserved |
| Tb927.3.4780 | hypothetical protein, conserved |
| Tb927.4.1020 | serine-palmitoyl-CoA transferase, putative |
| Tb927.4.1120 | hypothetical protein, conserved |
| Tb927.4.1160 | hypothetical protein, conserved |
| Tb927.4.1350 | glyoxalase II (glx2-2) |
| Tb927.4.2480 | 8-oxoguanine DNA glycosylase, putative |
| Tb927.4.3470 | hypothetical protein, conserved |
| Tb927.4.390 | hypothetical protein, conserved |
| Tb927.4.4910 | 3,2-trans-enoyl-CoA isomerase, mitochondrial precursor, putative |
| Tb927.4.5580 | variant surface glycoprotein (VSG, atypical), putative |
| Tb927.4.700 | hypothetical protein, conserved |
| Tb927.5.1390 | 64 kDa invariant surface glycoprotein (ISG64) |
| Tb927.5.1410 | 64 kDa invariant surface glycoprotein (ISG64) |
| Tb927.5.1430 | 64 kDa invariant surface glycoprotein (ISG64) |
| Tb927.5.1720 | hypothetical protein, conserved |
| Tb927.5.2030 | hypothetical protein, conserved |
| Tb927.5.2790 | mitochondrial DNA polymerase beta-PAK (Pol beta-PAK) |
| Tb927.5.309b | hypothetical protein |
| Tb927.5.310 | hypothetical protein |
| Tb927.5.4060 | hypothetical protein, conserved |
| Tb927.5.410 | hypothetical protein |
| Tb927.5.4670 | variant surface glycoprotein (VSG, atypical), putative |
| Tb927.5.4810 | variant surface glycoprotein (VSG, atypical), putative |
| Tb927.5.490 | methyltransferase, putative |
| Tb927.5.4950 | variant surface glycoprotein (VSG, atypical), putative |
| Tb927.5.5390 | Variant Surface Glycoprotein, putative |
| Tb927.5.5400 | Variant Surface Glycoprotein, putative |
| Tb927.5.5610 | Variant Surface Glycoprotein, putative |
| Tb927.6.1200 | hypothetical protein, conserved |
| Tb927.6.1730 | hypothetical protein, conserved |
| Tb927.6.2260 | hypothetical protein, conserved |
| Tb927.6.2320 | hypothetical protein, conserved |
| Tb927.6.2560 | hypothetical protein, conserved |
| Tb927.6.260 | hypothetical protein |
| Tb927.6.2890 | single strand-specific nuclease, putative |
| Tb927.6.3300 | GPI alpha-mannosyltransferase I (GPI14) |
| Tb927.6.450 | EP3-2 procyclin,PARP,procyclin PARP A,procyclin B1- alpha,procyclic acidic repetitive protein A.beta,procyclic form specific polypeptide B1-alpha precursor |
| Tb927.6.480 | EP3-2 procyclin,PARP A-beta,surface protein EP3-2,surface protein EP3-2 procyclin precursor,procyclic form specific polypeptide A-beta precursor |
| Tb927.6.510 | GPEET2 procyclin precursor,PARP A-alpha,procyclin A-alpha,procyclic form specific polypeptide A-alpha precursor |
| Tb927.6.520 | EP3-2 procyclin,PARP A-beta,surface protein EP3-3 procyclin precursor,procyclic form specific polypeptide A-beta precursor |
| Tb927.6.5550 | variant surface glycoprotein (VSG, atypical), putative |
| Tb927.7.1300 | protein disulfide isomerase, putative |
| Tb927.7.2190 | hypothetical protein, conserved |
| Tb927.7.2700 | NADH-cytochrome b5 reductase, putative (B5R) |
| Tb927.7.280 | cyclophilin-type peptidyl-prolyl cis-trans isomerase, putative (PPIase) |
| Tb927.7.3250 | expression site-associated gene (ESAG) protein, putative,expression site-associated gene 6 (ESAG6) protein, putative |
| Tb927.7.3260 | expression site-associated gene (ESAG) protein, putative,expression site-associated gene 7 (ESAG7) protein, putative |
| Tb927.7.3680 | ubiquitin/ribosomal protein S27a, putative |
| Tb927.7.470 | hypothetical protein |
| Tb927.7.4720 | hypothetical protein |
| Tb927.7.6670 | hypothetical protein, conserved |
| Tb927.7.7090 | hypothetical protein, conserved |
| Tb927.7.7420 | ATP synthase alpha chain, mitochondrial precursor,ATP synthase F1, alpha subunit |
| Tb927.7.7430 | ATP synthase alpha chain, mitochondrial precursor,ATP synthase F1, alpha subunit |
| Tb927.7.900 | hypothetical protein, conserved |
| Tb927.8.130 | variant surface glycoprotein (VSG, atypical), putative |
| Tb927.8.170 | variant surface glycoprotein (VSG, atypical), putative |
| Tb927.8.2140 | hypothetical protein, conserved |
| Tb927.8.3390 | hypothetical protein, conserved |
| Tb927.8.3540 | hypothetical protein, conserved |
| Tb927.8.3730 | hypothetical protein, conserved |
| Tb927.8.480 | phosphatidic acid phosphatase protein, putative |
| Tb927.8.5240 | hypothetical protein, conserved |
| Tb927.8.6600 | hypothetical protein, conserved |
| Tb927.8.7280 | hypothetical protein, conserved |
| Tb927.8.7530 | 3,2-trans-enoyl-CoA isomerase, mitochondrial precursor, putative |
| Tb927.8.8030 | hypothetical protein, conserved |
| Tb927.9.10130 | hypothetical protein, unlikely |
| Tb927.9.1050 | variant surface glycoprotein (VSG), putative |
| Tb927.9.10610 | hypothetical protein, unlikely |
| Tb927.9.10840 | hypothetical protein, conserved |
| Tb927.9.12730 | chaperone protein DNAj, putative |
| Tb927.9.1280 | variant surface glycoprotein (VSG, atypical), putative |
| Tb927.9.1300 | hypothetical protein, unlikely |
| Tb927.9.13200 | unspecified product |
| Tb927.9.14070 | short-chain dehydrogenase, putative |
| Tb927.9.16200 | variant surface glycoprotein (VSG, atypical), putative |
| Tb927.9.16250 | variant surface glycoprotein (VSG, atypical), putative |
| Tb927.9.17560 | hypothetical protein, unlikely |
| Tb927.9.1920 | hypothetical protein, conserved |
| Tb927.9.2260 | hypothetical protein, conserved |
| Tb927.9.440 | variant surface glycoprotein (VSG, atypical), putative |
| Tb927.9.4520 | mitochondrial processing peptide beta subunit, putative,metallo-peptidase, Clan ME, Family M16 |
| Tb927.9.4790 | hypothetical protein, unlikely |
| Tb927.9.570 | variant surface glycoprotein (VSG, atypical), putative |
| Tb927.9.5990 | hypothetical protein, unlikely |
| Tb927.9.7040 | hypothetical protein, conserved |
| Tb927.9.900 | variant surface glycoprotein (VSG), putative |
| Tb927.9.9810 | hypothetical protein, conserved |
| Tb927.9.9930 | hypothetical protein, unlikely |
