# Supplementary material for: An Essential Signal Peptide Peptidase Identified in an RNAi Screen of Serine Peptidases of Trypanosoma brucei
Source: PLoS One. 2015 Mar 27;10(3):e0123241. doi: 10.1371/journal.pone.0123241 (PMC4376731; doi:10.1371/journal.pone.0123241)
Supplement: S1 Fig — (DOCX) [file pone.0123241.s004.docx]

**Supporting Information Figure S2. Alignment of *SPPI* and recoded *SPPI* sequences**

10 20 30 40 50 60

SPP1 TGCTGCATCGGGCTGATGCGACGGCGCTGCGGATTTTGCGTGAGCAGCATGTGGAATCAT

::::::: :: :: ::::: :: ::::: :: :: : ::::::::::: :: ::

recode TGCTGCACCGTGCCGATGCTACTGCGCTTCGTATACTTCGTGAGCAGCACGTCGAGAGCC

70 80 90 100 110 120

SPPI TAGCCATGTCTGCACAAACAGGACCCATTAATTACATTAAAAAAACTTTTAAGGACATTG

: :::::: :: :: :: :: ::::: :: :: :: :: :::::::: :::::::: :

recode TTGCCATGAGCGCGCAGACTGGTCCCATCAACTATATCAAGAAAACTTTCAAGGACATAG

130 140 150 160 170 180

SPPI TTGATACAATGCCGGACAGACGCAACACCTTCTTAATGTTGGTAACTCTCTGCACATTCT

: ::::: :::::::: : ::::: ::::: : ::: : :: :: :: ::::: ::::

recode TAGATACGATGCCGGATCGGCGCAATACCTTTCTTATGCTTGTGACACTTTGCACCTTCT

190 200 210 220 230 240

SPPI TTGTTGGTTGGCGTACAGCCATCGCGGTGACCGATTGTGAAAGCCCACTTGTTGTGGTGC

:::: ::::::::::: :: :: :: :: :: ::::::::: :::: : :: :: ::::

recode TTGTCGGTTGGCGTACGGCAATTGCAGTTACAGATTGTGAATCCCCATTGGTCGTTGTGC

250 260 270 280 290 300

SPPI TCTCAGGAAGTATGGAACCCTTCATGTTTCGTGGTGACTTACTTGTTCTGCATAATATTG

: :: ::::: :::::::: :: ::::: :: :: ::: : :: ::::: ::::: :: :

recode TGTCTGGAAGCATGGAACCTTTTATGTTCCGGGGCGACCTCCTCGTTCTTCATAACATAG

310 320 330 340 350 360

SPPI GCGAACCCACGATGGGGGATGTCGTTGTTTTCTCTCTCCCCAACCGTACAATTCCAATCG

: :: :: :: ::::: ::::: :: :: :: : :: ::::: :::::::: :: :

recode GTGAGCCAACAATGGGCGATGTGGTAGTGTTTAGCTTGCCTAACCGCACAATTCCTATTG

370 380 390 400 410 420

SPPI TTCATCGCGTCCATCGCATTCGATTACTTGAGGACGGTGTGACTCGTTTGTACCTCACGA

:::::::::: :: :: :: :: : : :: :: :: :: :::::::::::::: :: :

recode TTCATCGCGTGCACCGTATCCGCCTTTTGGAAGATGGGGTTACTCGTTTGTACCTTACAA

430 440 450 460 470 480

SPPI AAGGGGATAACAATGAAATGGATGACCGTACATTATATCCGCGCGGATATCATTGGGTAG

: :: :: :: :: :::::::: ::::::::::: ::::: :: :: ::::: ::::: :

recode AGGGCGACAATAACGAAATGGACGACCGTACATTGTATCCACGGGGTTATCACTGGGTGG

490 500 510 520 530 540

SPPI AGAAAAAAGATATCATTGGGAAAGTTGCCGTGCTGGTGCCGCGGGTGGGTTTTATTACGC

: :: ::::: :::::::::::::: :: ::::: :: :: :: ::::: ::::::::::

recode AAAAGAAAGACATCATTGGGAAAGTGGCTGTGCTCGTACCACGCGTGGGGTTTATTACGC

550 560 570 580 590 600

SPPI TAATTGCCGAAGACCACTCATGGGCAAAACTTGTGCTTGTTCCACTGGCATTGATATGGT

: ::::: ::::: ::: ::::: :: ::::: :: :: :: :::::: : :: ::::

recode TCATTGCGGAAGATCACAGCTGGGCGAAGCTTGTTCTGGTCCCGCTGGCACTCATTTGGT

610 620

SPPI GCTGGTACACCGGAATGTAA

::::::: :: :: ::::::

recode GCTGGTATACGGGGATGTAA
